# CAEVT: Convolutional Autoencoder Meets Lightweight Vision Transformer for Hyperspectral Image Classification

**DOI:** 10.3390/s22103902

**Published:** 2022-05-20

**Authors:** Zhiwen Zhang, Teng Li, Xuebin Tang, Xiang Hu, Yuanxi Peng

**Affiliations:** 1 The State Key Laboratory of High-Performance Computing, College of Computer, National University of Defense Technology, Changsha 410073, China; zhangzhiwen20@nudt.edu.cn (Z.Z.); xbtang@nudt.edu.cn (X.T.); huxiang@nudt.edu.cn (X.H.); 2 Beijing Institute for Advanced Study, National University of Defense Technology, Beijing 100020, China; liteng09@nudt.edu.cn; 3 College of Advanced Interdisciplinary Studies, National University of Defense Technology, Changsha 410073, China

**Keywords:** convolutional neural network, autoencoder, vision transformer, hyperspectral image classification

## Abstract

Convolutional neural networks (CNNs) have been prominent in most hyperspectral image (HSI) processing applications due to their advantages in extracting local information. Despite their success, the locality of the convolutional layers within CNNs results in heavyweight models and time-consuming defects. In this study, inspired by the excellent performance of transformers that are used for long-range representation learning in computer vision tasks, we built a lightweight vision transformer for HSI classification that can extract local and global information simultaneously, thereby facilitating accurate classification. Moreover, as traditional dimensionality reduction methods are limited in their linear representation ability, a three-dimensional convolutional autoencoder was adopted to capture the nonlinear characteristics between spectral bands. Based on the aforementioned three-dimensional convolutional autoencoder and lightweight vision transformer, we designed an HSI classification network, namely the “convolutional autoencoder meets lightweight vision transformer” (CAEVT). Finally, we validated the performance of the proposed CAEVT network using four widely used hyperspectral datasets. Our approach showed superiority, especially in the absence of sufficient labeled samples, which demonstrates the effectiveness and efficiency of the CAEVT network.

## 1. Introduction

Remote sensing images contain abundant spectral and spatial information [1]; thus, numerous studies have been conducted on remote sensing images, such as land cover mapping [2], water detection [3], and anomaly detection [4]. HSI plays an indispensable role within the remote sensing community [5] and is widely used in change area detection [6], atmospheric environment research, vegetation cover detection [7], and mineral mapping [8]. However, the correlation between spectral bands is complex, which causes information redundancy while forming the curse of dimensionality. In addition, the analysis and processing of HSI require a large amount of computation; therefore, it is essential to reduce the computation overloadwhile maintaining processing accuracy.

Principal component analysis (PCA) [9] and linear discriminant analysis (LDA) [10] are classical dimensionality reduction methods. However, these linear methods cannot handle the nonlinear distribution of spectral vectors well. Following the successful application of deep learning in various fields, this technology has also attracted much attention for use in dimensionality reduction. Deep learning has a strong nonlinear processing ability, in which the use of autoencoders is a typical unsupervised learning method. Zhang et al. [11] introduced a basic framework for the application of deep learning to remote sensing data processing and proposed a stacked autoencoder for data dimensionality reduction. To fully extract the rich spatial–spectral information, Ma et al. [12] proposed a spatial update deep autoencoder, which is based on a deep encoder with added regularization terms. Ji et al. [13] proposed a three-dimensional (3D) convolutional autoencoder for the construction of a 3D input using spatial neighborhood information. However, these models are all followed by a simple classification model after the use of the autoencoder for feature extraction, which leads to the problem of insufficient feature extraction. Therefore, we hoped to further explore deep learning methods for HSI classification to fully extract feature information and finally achieve higher performances.

In recent years, CNNs have been proven to be outstanding for image recognition, speech recognition, and pattern analysis. However, CNNs are vulnerable to backdoor attacks. Some outstanding works have endeavored to solve this problem, such as MedicalGuard [14], BlindNet backdoor [15], the multi-model selective backdoor attack method [16], and the use of a de-trigger autoencoder against backdoor attacks [17]. CNN-based methods have been widely used for image processing and also for HSI classification tasks. These methods have achieved significant breakthroughs due to their local processing and shared weight properties. According to the extracted features, these models can be divided into three categories: spectral-based methods, spatial-based methods, and spatial–spectral cooperative methods. The spectral-based methods classify each pixel by making use of the rich spectral information. Mu et al. [18] proposed a dual-branch CNN-based method for multispectral entropy super-pixel segmentation for HSI classification. Yang et al. [19] proposed a deep similarity network to solve imbalances between the slight intra-category and large inter-category differences. Moreover, a new pixel similarity measurement method has been developed using a double-branch neural network to deal with the task of classification. In an attempt to ameliorate the problem of mixed pixels destroying the credibility of original spectral information and the computational efficiency of overly complex models, Gao et al. [20] proposed a 3D data preprocessing method and designed a new sandwich CNN that is based on the proposed method. To improve the performance of HSI classification that is based on spectral feature learning, a dual-channel attention spectral feature fusion method was proposed, based on a CNN, which extracts local and inter-block spectral features simultaneously in a parallel manner after grouping the adjacent spectral bands [21]. The spatial-based methods only use spatial information, which means that the rich spectral information is not used. A consolidated CNN [22] was proposed to overcome the problem of insufficient spatial resolution. Fang et al. [23] proposed a 3D asymmetric inception network to overcome this overfitting problem. The third group of methods extracts spatial and spectral information at the same time and then fuses the extracted information for HSI classification. Sun et al. [24] developed a method for extracting local features and then concatenating the spatial and spectral features for classification. Zhao et al. [25] constructed an architecture that is based on a spatial–spectral residual network for deep feature extraction.

Although CNNs have achieved efficient performances in HSI classification, two main problems still exist. On the one hand, HSI classification comprises point-wise prediction, so the convolutional kernels cannot extract all of the useful information due to different regional topographies. On the other hand, the size of the convolutional kernels limits the receptive field of a CNN, which makes it impossible to carry out long-range modeling. The use of transformers [26] makes up for this deficiency.

Along with the rapid development of deep learning, CNNs have always been mainstream in the computer vision (CV) field and have demonstrated some extraordinary achievements. Correspondingly, transformers have dominated the natural language processing field. Since 2020, transformers have started to be used in the CV field, such as for image classification (ViT, DeiT, etc.) [27,28], target detection (DETR, deformable DETR, etc.) [29,30], semantic segmentation (SETR, MedT, etc.) [31,32], and image generation (GANsformer) [33]. For CV problems, convolution has a number of natural advantages, such as translation equivalence and locality. Although transformers do not have the above-mentioned advantages, they can obtain long-range information and extract global information that is based on their unique structure. By contrast, CNNs need to continuously accumulate convolutional layers to obtain larger receptive fields. Based on a ViT, Li et al. [34] proposed a simple yet effective visual transformer (ViT) called SimViT, which uses multi-head central self-attention and a simple sliding window to concentrate the spatial structure and local information into the ViT. Simultaneously, multi-scale hierarchical features can be applied to various intensive visual prediction tasks. Given the wide application of transformers within the CV field, some studies have introduced ViTs into HSI classification. Hong et al. [35] examined the problem of HSI classification from the perspective of sequencing and proposed SpectralFormer, which applies a transformer to HSI classification without convolution or cyclic units. He et al. [36] proposed a spatial–spectral transformer for HSI classification, which uses a well-designed CNN to extract features and adopts a densely connected transformer to deal with the long-range dependencies. Qing et al. [37] improved transformers to enable them to extract the spectral–spatial features of HSIs by utilizing the spectral attention and self-attention mechanisms. However, these models are still heavyweight, which leads to low efficiency.

As CNNs use the natural inductive bias advantage to learn visual representation information, they can only establish local dependencies in the spatial information domain. A ViT that is based on the self-attention mechanism can capture the global receptive field of the input feature map and can establish global dependencies in the spatial dimension to learn the global visual representation information. However, due to the structure of the self-attention mechanism, network architectures usually have a large number of parameters and computations. In view of this, we committed to combining the advantages of CNNs and ViTs into the design of an efficient network architecture. Moreover, the feature destruction that is caused by the linear dimensionality reduction method was also a point of our concern. In this study, we adjusted the structure of the MobileViT [38] and constructed a lightweight, robust, and high-performance framework, which can adapt to HSI processing. The proposed method combines the advantages of CNNs and ViTs and improves previous classification performances. Finally, we conducted experiments using four benchmark hyperspectral datasets to confirm the feasibility and excellence of our method for HSI classification.

The three significant contributions of this paper are as follows:

(a) According to our review of the literature, this study is the first to attempt to extend a lightweight ViT (MobileViT) for HSI classification. The MobileViT network can extract local and global information simultaneously and promote accurate classification;

(b) To preserve the more original information of HSI while reducing computational costs, we chose an end-to-end 3D convolutional autoencoder (3D-CAE) network for nonlinear feature dimensionality reduction. Moreover, we proposed an efficient end-to-end CAEVT network, which is based on the MobileViT and the 3D-CAE network;

(c) We evaluated the proposed method using four public datasets and achieved excellent classification results compared to other classification algorithms. In addition, sufficient ablation experiments demonstrated that the proposed method is efficient and effective in terms of time consumption, the number of parameters, and floating point operations (FLOPs). It is worth nothing that our CAEVT network also achieves a competitive performance when labeled samples are scarce.

The rest of this article is organized as follows. Section 2 introduces the experimental datasets and the proposed framework. The experimental results and an analysis of different methods are presented in Section 3 and Section 4, respectively. Finally, Section 5 presents the conclusions.

## 2. Datasets and Methods

In this section, we introduce the four public HSI datasets that were used in this study and the proposed CAEVT network in detail.

### 2.1. Introduction: Datasets

This study used four common HSI datasets to compare and verify the proposed method: the Indian Pines (IP) dataset (Table 1), Salinas (SA) dataset (Table 1), Pavia University (PU) dataset (Table 2), and Houston (HS) dataset (Table 2).

The PU dataset comprises the continuous imaging of 115 bands within the wavelength range of 0.43–0.86 μm, of which 12 bands were eliminated due to noise, and the spatial resolution of the images is 1.3 m. The size of the data points is 610 × 340, including 42,776 feature pixels in total. These pixels contain nine types of ground truths, including trees, asphalt roads, bricks, pastures, etc.

The IP dataset contains images with a spatial dimension of 145 × 145 pixels and 224 spectral bands within the wavelength range of 0.4–2.5 μm, of which 24 spectral bands that encompassed water absorption areas were deleted. There are 10,249 accessible ground truths, which are divided into 16 vegetation classifications.

The SA dataset comprises the continuous imaging of 224 bands, 20 of which were eliminated because they could not be reflected by water. The spatial resolution of the images is 3.7 m. The size of the data points is 512 × 217 and 54,129 pixels can be applied to the classification. These pixels are divided into 16 categories, including fallow, celery, etc.

The HS dataset was developed for the 2013 IEEE GRSS data fusion competition. The data point size is 349 × 1905, including 144 bands with a spectral range of 364–1046 nm. The ground truths are labeled into 15 categories.

### 2.2. Three-Dimensional Convolutional Autoencoder

The use of an autoencoder is an effective way to extract deep-seated features due to its hierarchical structure. For a given autoencoder, our goal was to obtain the same output as the input, as far as possible, by optimizing the parameters. Naturally, we obtained several different representations of input *X* (the feature maps of each layer represent the different representations).

An autoencoder has two parts: An encoder and a decoder. Furthermore, a loss function is required to measure any loss. The smaller the loss, the closer the obtained features are to the features of the original input data. The parameters of the encoder and decoder can be adjusted by optimizing the loss function. In this study, to extract spatial–spectral features simultaneously, we used a 3D-CAE (Equation (1)) to construct the encoder and decoder:

(1) v=σ(W∗X+b)

where *W* represents the convolutional kernel, *X* is the input, *b* is the bias, σ is the activation function, and *v* is the extracted features.

**Figure 1:**
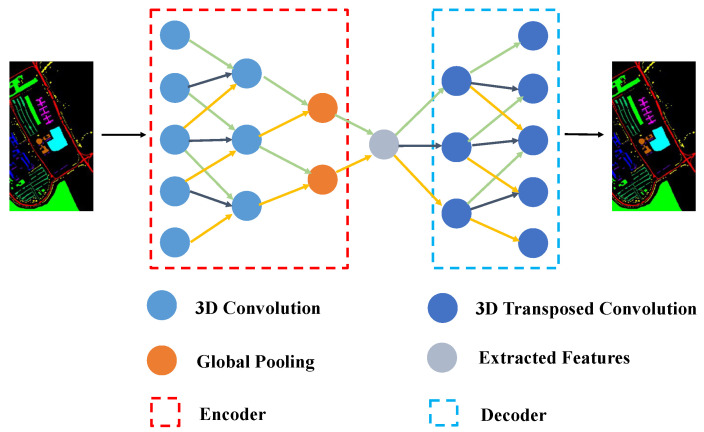
Structure of the 3D-CAE model.

The structure of the 3D-CAE is shown in Figure 1. The encoder part comprises convolutional and pooling layers: two convolutional layers and an average pooling layer. Similarly, the decoder consists of two deconvolutional layers. The convolutional layers are used for local processing and the pooling layer is used for downsampling. The deconvolutional layers are used to reconstruct information. The results are measured by the following equation:

(2) $$L={∥X^{\prime }-X∥}^{2}$$

where $X^{\prime }$ represents the reconstructed image, *X* represents the input image, and *L* stands for the loss. The smaller the *L* value, the closer the reconstructed features are to the features of the input image.

In addition, a normalization operation [39] (Equation (3)) and activation function (Equation (4): PReLU [40]) were added to speed up propagation and alleviate overfitting.

(3) $$\bar{X}=\frac{X-E\left(X\right)}{\mathrm{Var}\left(X\right)}$$

(4) $$\mathrm{PReLU}\left(x_{i}\right)=\left\{\begin{array}{ccc} x_{i} & \;\mathrm{if}\; & x_{i}>0\\ a_{i}x_{i} & \;\mathrm{if}\; & x_{i}\leq 0 \end{array}\right.$$

where $a_{i}$ is the artificial set and $x_{i}$ stands for the input. The activation function can increase nonlinearity in the lower dimensions, but it may destroy spatial characteristics in the higher dimensions [41]. We verified this through the experiments that are detailed in Section 4.1. So, we did not choose to adopt any activation functions in the last deconvolutional layer.

Taking the PU dataset as an example, the parameters of the 3D-CAE that was developed in this study are listed in Table 3. We used larger cores for the spectral channels to rapidly reduce the number of bands. The mean squared error (MSE) loss function was used to measure the deviation between the reconstructed data and the original data. The adaptive moment estimation (Adam) method was adopted to optimize the network parameters. In addition, we set the learning rate to 0.001. Finally, the obtained features were transmitted into the next structure.

### 2.3. Vision Transformer

The transformer encoder consists of an alternating multi-head self-attention layer and a multi-layer perceptron (MLP) block. First, the input feature is mapped into Query (*Q*), Key (*K*), and Value (*V*) using the MLP. Next, the encoder is gained according to the following expression:

(5) $$Attention\left(Q,K,V\right)=\mathrm{softmax}\left(\frac{QK^{T}}{\sqrt{d_{k}}}\right)V$$

(6) $$\begin{array}{c} MultiHead\left(Q,K,V\right)=Concat\left(head_{1},\ldots ,head_{h}\right)W^{O} \end{array}$$

(7) $$head_{i}=Attention\left(QW_{i}^{Q},KW_{i}^{K},VW_{i}^{V}\right)$$

The $head_{i}$ expression calculates its own attention and then multiplies it by $W^{O}$ to obtain the aggregate feature representation.

Inspired by the successful scaling of the transformer in NLP, we developed a ViT that tries to directly explore the standard transformer in the image and reduces the amount of modification as much as possible. To this end, the image is split into patches and the linear embedding sequence of these image blocks is then used as the input for the transformer.

**Figure 2:**
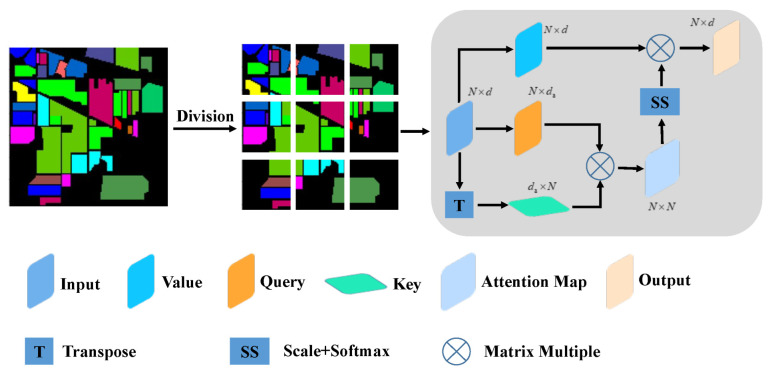
Structure of the self-attention mechanism.

The standard transformer accepts a one-dimensional sequence of token embedding as its input. In order to process 2D images, the ViT reshapes the image $X\in R^{H\times W\times C}$ into a flattened 2D sequence $x_{p}\in R^{N\times \left(P^{2}\cdot C\right)}$, where (*H*, *W*) is the resolution of the original image, *C* is the number of channels (RGB image, *C* = 3), (*P*, *P*) is the resolution of each image block, $N=HW/P^{2}$ is the number of generated image blocks, and *N* is the effective input sequence length of the transformer. Later, we demonstrate how we developed this transformer for HSI processing (Figure 2).

### 2.4. MobileViT Block

In CNNs, locality, 2D neighborhood structures, and translation equivalences exist within each layer of the model; however, ViTs have much less image-specific inductive bias than CNNs. In ViTs, the MLP layers are local and equivariant, yet the self-attention layers are global. As an alternative to the original image blocks, the input sequences can be composed of CNN feature maps. Based on the above considerations, this model was proposed in the literature [38].

**Figure 3:**
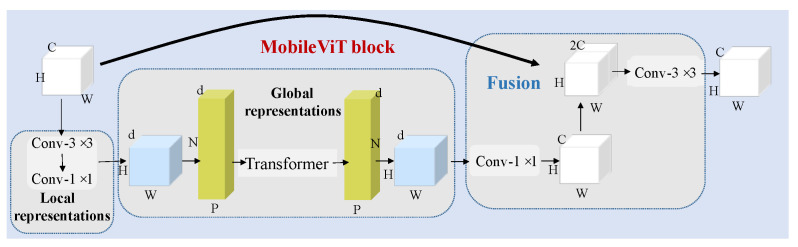
Structure of the MobileViT block.

The MobileViT block is shown in Figure 3. It is assumed that the input character is $X_{0}\in R^{H\times W\times C}$. Then, the local expression can be obtained using convolution. At this stage, a separable convolutional structure with convolutional kernels of 3 × 3 and 1 × 1 is used to replace the normal convolution. The separable structure can easily change the number of channels and speed up the operation. The resulting characteristic is recorded as $X_{1}\in R^{H\times W\times d}$ (d<C). Due to the heavyweight peculiarity of the ViT, we reduced the input features to a lower *d* dimension. As the ViT operates, the input feature map is divided into a series of disjointed blocks, which are recorded as $X_{3}\in R^{N\times P\times d}$. Under these conditions, *h* and *w* were the input parameters, which were to 2, and P=h×w.

For each p∈{1,⋯,P}, the transformer is used to achieve global processing and the relationship between each patch is also obtained. The expression is as follows:

(8) $$X_{4}\left(p\right)=Transformer\left(X_{3}\left(p\right)\right),1\leq p\leq P$$

Then, the size of the feature, which is recorded as $X_{5}\in R^{H\times W\times C}$, is reconstructed to be the same as that of the initial image. Low-level features $X_{1}$ and high-level features $X_{5}$ are combined in the third dimension. Next, the dimension is reduced to *C* using a convolution with a kernel of 3 × 3. In addition, the parameters of the MobileViT block are listed in Table 4. This contains all of the details about the MobileViT block.

### 2.5. The Framework of the Proposed CAEVT

**Figure 4:**
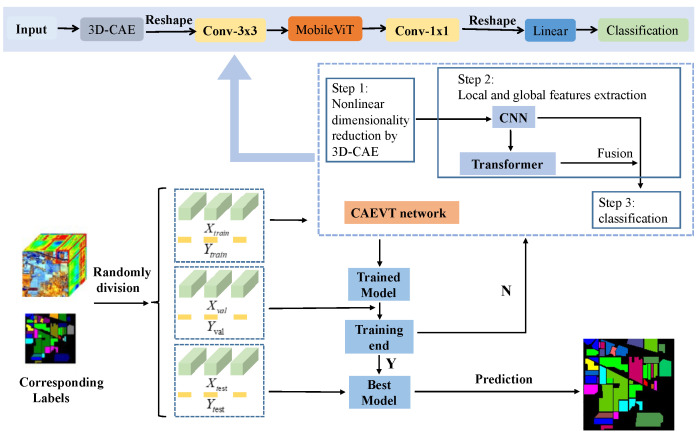
Flowchart of the proposed procedure.

The framework contains three steps: dataset generation, training and validation, and prediction, which can be seen in Figure 4. First of all, the dataset is randomly divided into a training set, validation set, and testing set. For the training set, four channels (*C*, *B*, *H*, and *W*) are reshaped into three channels (CB, *H*, and *W*) (*C* stands for the channel and *B* stands for the band) after using the 3D-CAE model to reduce the dimensions. Next, a convolutional layer is adopted and the features are input into the MobileViT block for the extraction of local and global features. Before the features are input into the classification network, another convolutional layer, an average pooling layer, and a dropout rate of 0.2 are adopted. Afterward, the features are reshaped into one dimension for classification. The classification network consists of a fully connected layer. Finally, a cross-entropy loss function is adopted to calculate the error.

Taking the PU dataset as an example, the CAEVT network is shown in Figure 4 and the parameters are listed in Table 5. In addition, all strides and paddings in the convolutions were set to 1.

In the previous literature, spatial information is captured by learning the linear relationship between patches and considering that CNNs can extract local properties and transformers can obtain global properties. The CAEVT network adopts convolutions and a transformer to capture spatial information. The steps of the proposed CAEVT network are summarized in Algorithm 1. Within this framework, the MobileViT can be iterated to improve accuracy at the cost of computation time; however, the block was only adopted once in this study for the sake of efficiency. In addition, we illustrate the lightweight nature of the CAEVT network by comparing the FLOPs and the number of parameters in Section 4.2.
**Algorithm 1:** The proposed method.Input: HSI original data *X* and label *Y*;Output: The evaluation index.(1) Divide randomly the input data *X* and annotated label *Y* into training set ($X_{train},Y_{train}$), validation set ($X_{val},Y_{val}$), and test set ($X_{test}$,$Y_{test}$).(2) Optimize CAEVT network using training set ($X_{train},Y_{train}$).(3) Estimate the model using validation set ($X_{val},Y_{val}$).(4) Judge whether the training is over. If yes, output the optimal model; if not, continue the training.(5) Save the optimal model after training 50 epochs.(6) Input $X_{test}$ to obtain the predicted result and calculate the evaluation index.

### 2.6. Experimental Settings

The following four methods were compared to the proposed method.

SSRN [42]: Based on the 3D convolutional classification models that were proposed by our predecessors, the idea of a skip connection for ResNet [43] was introduced. This network uses spectral residual blocks and spatial residual blocks to extract rich spectral and spatial features.

FDSSC [44]: Using different convolutional kernel sizes to extract spectral and spatial features and using an effective convolutional method to reduce the high dimensions, an end-to-end fast dense spectral–spatial convolutional network for HSI classification was proposed.

DBMA [45]: A double-branch multi-attention mechanism network for HSI classification was proposed. The network uses two branches, which adopt attention mechanisms, to extract spectral and spatial features and reduce the interference between the two types of features. Finally, the extracted features are fused for classification.

DBDA [46]: Based on DBMA, a network was designed, namely a double-branch dualattention mechanism network, for HSI classification. This method further enhances the ability of the network to extract spectral and spatial features and has a better performance when there are limited training samples.

We executed the public code of these algorithms to obtain our results. The accuracy was measured using the three metrics of overall accuracy (OA), average accuracy (AA), and kappa coefficient. OA represents the proportion of correctly predicted samples out of the total number of samples. The average accuracy of all categories is denoted by AA. The consistency between the ground truth and a result is shown by the kappa coefficient. The better the categorization results, the higher the three metric values. Additionally, all experiments were carried out within the framework of Pytorch 1.10.2 using the RTX Titan GPU.

## 3. Results

In this section, experiments on four popular datasets were executed to compare the accuracy and efficiency of the proposed algorithm to those of the other methods. We divided the dataset into three parts: the training set, validation set, and testing set. Due to the limited number of annotated samples in the IP and HS datasets, 5% of the samples were randomly selected each for training and validation. For the PU and SA datasets, the proportion of samples for training and validation was set to 1%. Furthermore, in the proposed algorithm, the learning rate was set to 0.001 and the weight decay was set to 0.0005. The parameters of the algorithms for comparison were based on their best settings, which were provided by the relevant authors. Finally, the number of training epochs for all algorithms was set to 50.

### 3.1. Results for the IP Dataset

**Figure 5:**
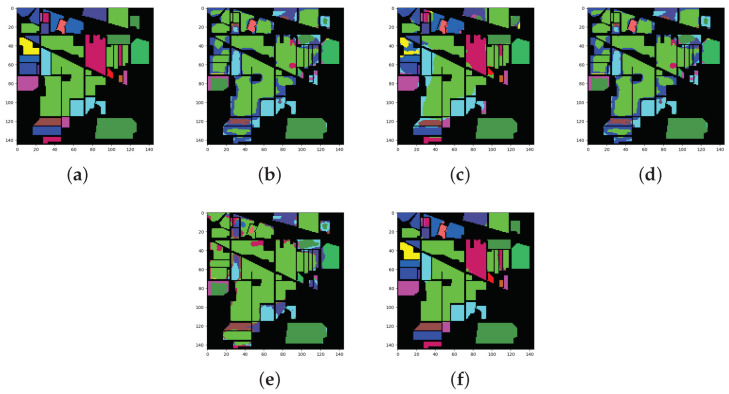
Classification maps for the IP dataset: (**a**) ground truth (GT); (**b**–**f**) results from the different algorithms. (**a**) GT; (**b**) SSRN; (**c**) FDSSC; (**d**) DBMA; (**e**) DBDA; (**f**) Proposed.

The classification results of all methods when using 5% of the data for training samples are shown in Table 6 and the best results are shown in bold. The ground truth and prediction maps of the methods are shown in Figure 5.

The main characteristic of the IP dataset is that the number of labeled samples is small and the data distribution is imbalanced. In particular, the number of samples in class 1, class 7, class 9, and class 16 is less than 100, which is far less than that in the other classes. The SSRN algorithm absorbed the characteristics of the ResNet algorithm and performed the best out of the four algorithms that were adopted for comparison. This algorithm achieved optimal results for class 2, class 4, class 6, class 8, class 13, class 14, and class 16. Notably, the accuracy of class 4 and class 16 was 100%. The DBMA algorithm achieved the worst results, with 53.49% OA, 40.92% AA, and 44.91% Kappa. For the DBDA algorithms with the attention mechanism, the results were not satisfactory. The DBDA algorithm used more attention mechanisms than the DBMA algorithm, so the former performed better than the latter. The results increased by 18.17% for OA, 16.22% for AA, and 21.93% for Kappa. The FDSSC and DBMA algorithms showed the best performance for class 16 and class 10, respectively. Additionally, the classification results from the other methods for class 1, class 7, and class 9 were 0, which we speculate was caused by the insufficient number of labeled samples. Similar to the SSRN algorithm, the proposed method obtained the best results for seven categories and surpassed the SSRN algorithm by a slim margin. Moreover, the network that we designed showed the best performance, with 90.71% OA, 78.61% AA, and 89.37% Kappa. It can also be observed from the prediction maps that the category boundaries that were obtained using the proposed method were more obvious and that the edges were clearer.

### 3.2. Results for the SA Dataset

**Figure 6:**
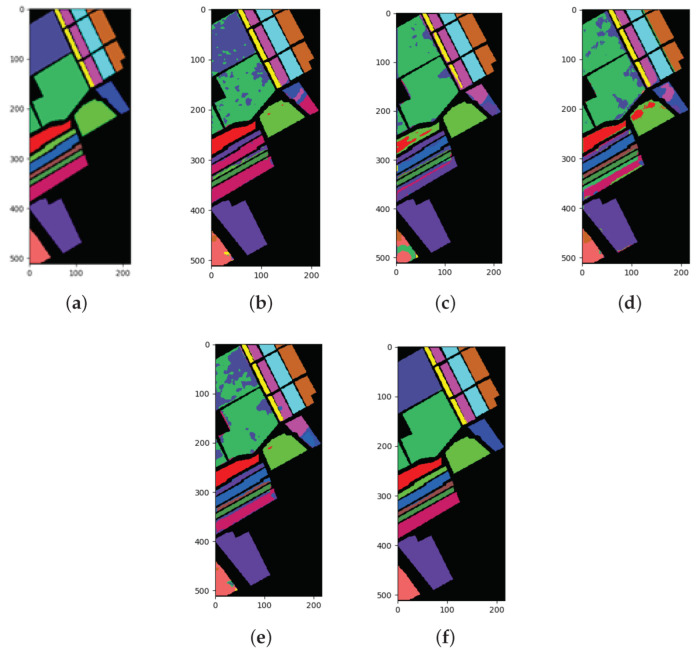
Classification maps for the SA dataset: (**a**) ground truth (GT); (**b**–**f**) results from the different algorithms. (**a**) GT; (**b**) SSRN; (**c**) FDSSC; (**d**) DBMA; (**e**) DBDA; (**f**) Proposed.

The classification results of all methods when using 1% of the data for training samples are listed in Table 7 and the best results are shown in bold. The ground truth and prediction maps of the methods are shown in Figure 6.

The main characteristics of the SA dataset are a large number of labeled samples and the balanced distribution of classes. For the SA dataset, the SSRN algorithm was error-free for class 6, class 13, and class 16. Similarly, the FDSSC algorithm was error-free for class 1, class 13, and class 16. In addition, a zero error was achieved by the DBMA algorithm for class 1 and by the DBDA algorithm for class 2, class 6, class 14, and class 16. Moreover, the proposed method achieved the best performance for class 3, class 4, class 5, class 7, class 9, class 10, class 11, class 12, and class 15. Compared to the FDSSC algorithm, which achieved the worst results, our proposed method improved by 27.45% for OA, 39.46% for AA, and 31.18% for Kappa. As shown in Table 7, the results from the CAEVT network were optimal, according to the three selected indexes, and the accuracy of each category that was classified using our method exceeded 89%. It can be observed from the prediction maps that the four methods that were adopted for comparison had some obvious misclassifications. The results that were obtained by the CAEVT network were consistent with the ground truth.

### 3.3. Results for the PU Dataset

**Figure 7:**
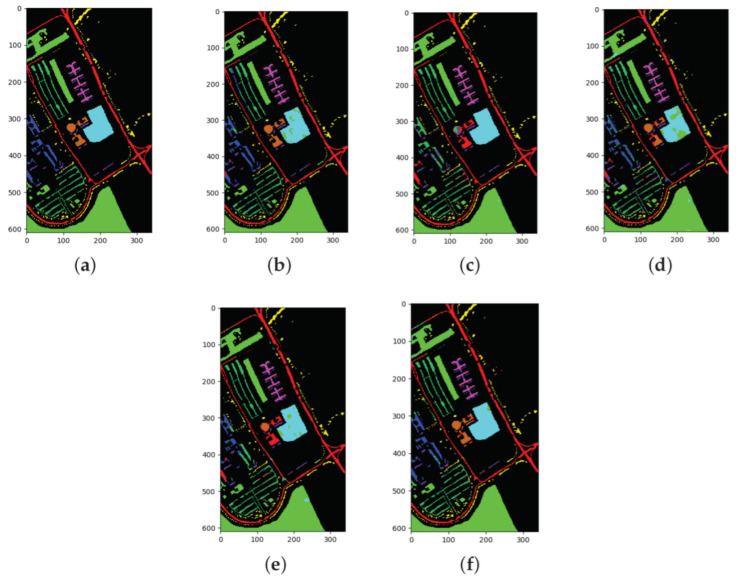
Classification maps for the PU dataset: (**a**) ground truth (GT); (**b**–**f**) results from the different algorithms. (**a**) GT; (**b**) SSRN; (**c**) FDSSC; (**d**) DBMA; (**e**) DBDA; (**f**) Proposed.

The classification results of all methods when using 1% of the data for training samples are listed in Table 8 and the best results are in bold. The ground truth and prediction maps of the methods are shown in Figure 7.

In the PU dataset, the SSRN algorithm demonstrated certain advantages and performed the best for class 1, class 2, and class 5. The performances of the FDSSC, DBMA, and DBDA algorithms were similar and were inferior to that of the SSRN algorithm. The proposed algorithm performed the best for class 4, class 5, class 6, and class 8. In addition, the proposed algorithm exceeded the SSRN algorithm by 0.24% for OA, 0.13% for AA, and 0.29% for Kappa. The other methods showed satisfactory accuracies for every category due to the sufficient number of samples. Moreover, we had difficulty observing any obvious differences between the prediction maps, which was a phenomenon that we speculate occurred due to the similar OAs.

The overall sample size of the PU dataset is large and basically balanced. Among them, class 1 and class 8 are the two classes with the largest number of samples, which far exceed the other classes.

### 3.4. Results for the HS Dataset

**Figure 8:**
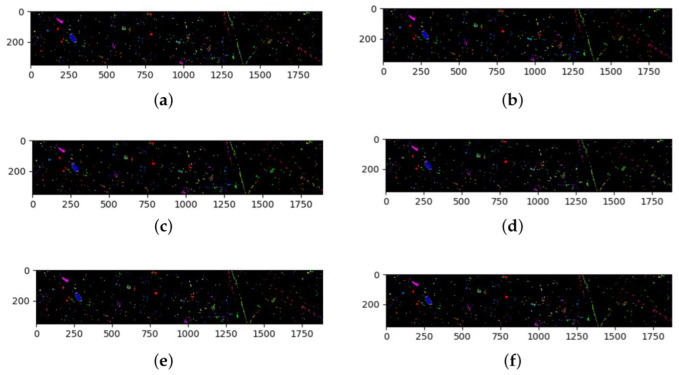
Classification maps for the HS dataset: (**a**) ground truth (GT); (**b**–**f**) results from the different algorithms. (**a**) GT; (**b**) SSRN; (**c**) FDSSC; (**d**) DBMA; (**e**) DBDA; (**f**) Proposed.

The classification results of all methods when using 5% of the data for training samples are listed in Table 9 and the best results are shown in bold. The ground truth and prediction maps of the methods are shown in Figure 8.

The overall sample size of the HS dataset is small and slightly imbalanced. Similar to the results from the SA dataset, the CAEVT network performed the best for nine classes. There was no problem of sample size imbalance and all methods performed well using this dataset. Among the contrast algorithms, the OA, AA, and Kappa of the SSRN algorithm were higher than those of the others but our proposed algorithm obtained the best results with 92.67% for OA, 90.78% for AA, and 92.06% for Kappa, as seen in Table 9. As seen in Figure 8, the proposed algorithm performed the best.

## 4. Discussion

In this section, a further analysis of the CAEVT network is provided. First, we compared the training and testing times, FLOPs, and the number of parameters to illustrate the lightweight nature of the proposed network. Second, the results of the ablation experiments confirmed the effectiveness of the 3D-CAE and MobileViT model. Finally, different proportions of training samples were input into the network and the results showed that the proposed algorithm could maintain its effectiveness, especially when the number of labeled samples was severely limited.

### 4.1. Selection of Activation Function

We proposed not to adopt any activation functions in the last layer in order to achieve better results, as described in Section 2.2. Taking the PU dataset as an example, we compared the results from using the PReLU, tanh, and sigmoid functions (Table 10). The data showed that an excellent performance could be obtained without using any activation functions.

### 4.2. Lightweight and Low-Latency Network

The aforementioned experiments showed that our algorithm could achieve a higher accuracy than the other algorithms that were compared in this study. Nevertheless, a good algorithm should balance accuracy with efficiency.

**Figure 9:**
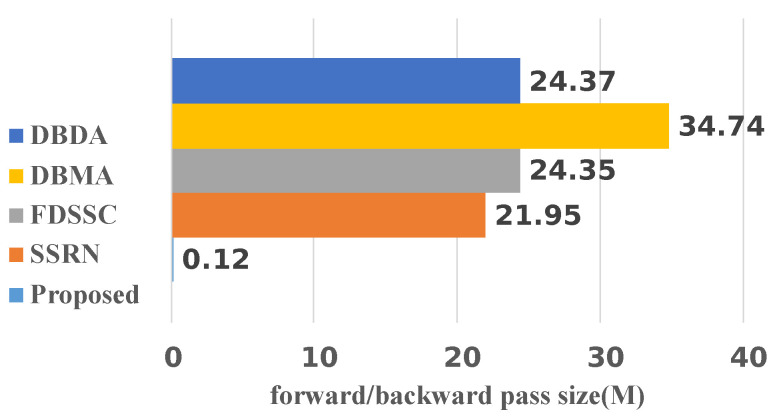
Size comparison of the models.

Our proposed method adopts convolution and a transformer to learn local and global representations. However, the transformer architecture usually has a large number of parameters, which results in a slow calculation speed, and the CNN also consumes a lot of time for the local processing. Thus, we counted the training times for 50 epochs and testing times of the algorithms (Table 11, Table 12, Table 13 and Table 14). Meanwhile, the forward–backward pass sizes of the five algorithms are shown in Figure 9.

**Figure 10:**
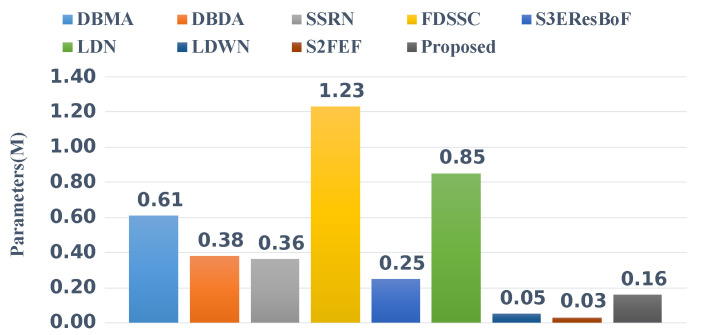
Parameters of the different networks.

**Figure 11:**
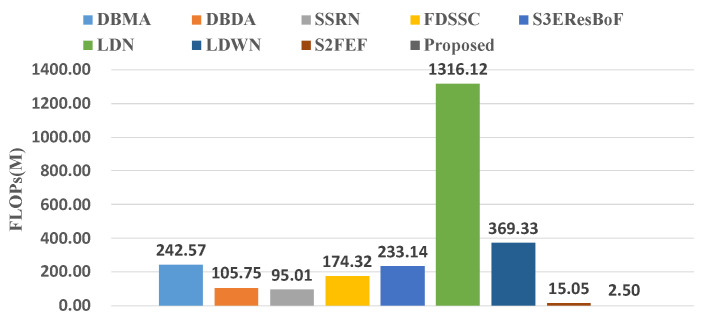
FLOPs of the different networks.

For comparison, we added a further four recently published methods that are committed to building lightweight networks. These four networks were: S3EResBoF [47], LDN [48], LDWN [49], and S2FEF [50]. The comparisons of the number of parameters and FLOPs are presented in Figure 10 and Figure 11. Out of the compared algorithms, the FDSSC had the most parameters and the number of parameters was approximately eight times that of our proposed method. The S2FEF possessed the lowest number of parameters out of the compared algorithms and the number of parameters was one fifth of that of the CAEVT network. Among the nine algorithms, our proposed algorithm had the smallest FLOP values. In comparison, the FDSSC algorithm took the longest time for training, followed by the SSRN algorithm. The time consumption of the DBMA and DBDA algorithms was similar, which was approximately twice that of the proposed method. As previously mentioned, the network that we built is lightweight and contains fewer parameters than other algorithms in the training process, so the training time was the shortest. However, the model parameters were not optimized in the testing process, so the testing time became the longest. Considering the training and testing times simultaneously, we consider the delay to be feasible. To sum up, the CAEVT is a lightweight network.

### 4.3. Effectiveness of the 3D-CAE

**Figure 12:**
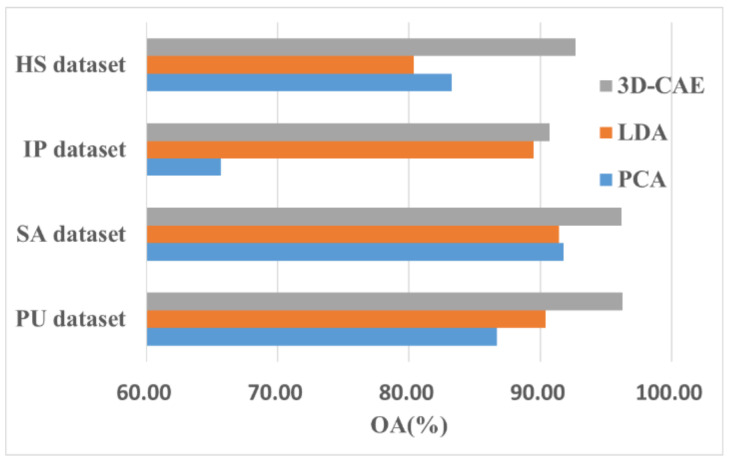
Effectiveness of the 3D-CAE.

To prove the effectiveness of the 3D-CAE model for HSI classification, we processed the data using PCA and LDA as a comparative study. Next, the extracted features were classified by the MobileViT. The results are shown in Figure 12.

We used different methods to reduce the dimensions to lower levels. For the IP and PU datasets, the LDA method was better than the PCA method, whereas for the HS and SA datasets, the results were the opposite. However, these results were not as good as those that were obtained by the 3D-CAE model. In addition, the experimental results using the PCA method were closest to those that were obtained by the 3D-CAE using the IP dataset. It can be observed from Figure 12 that the 3D-CAE method improved the classification accuracy for the four datasets and we can infer that the 3D-CAE adopts a nonlinear strategy to reduce the dimensions of the initial data and retain more of the original information.

### 4.4. Effectiveness of the MobileViT

**Figure 13:**
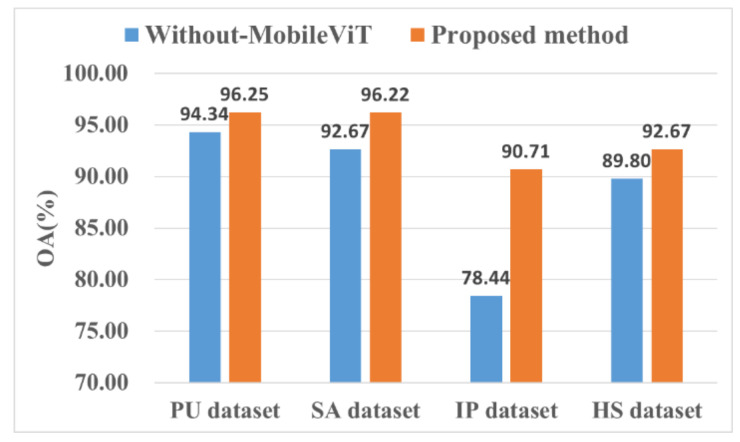
Effectiveness of the ViT.

Figure 13 shows the impact of using the MobileViT for performance improvement. We also tested a CNN without a MobileViT structure for comparison.

We observed that the global representation ability of the MobileViT enabled the model to learn more features than the other algorithms (Figure 13). For the IP dataset, the performance improvement was the most obvious, with the OA increasing by 12.27%, which was 1.94%, 3.55%, and 2.87% higher compared to the other three datasets. The experimental results of the four datasets showed that adding a MobileViT block to the network improved their performance.

### 4.5. Exploration of the Sample Proportions

Although deep learning-based algorithms have shown excellent HSI classification performances, they usually need a large number of training samples and network parameters and also have higher computational costs. For HSI classification, the number of available labeled pixels is usually very limited due to the difficulty of collection and the cost of labeling. Therefore, we explored the impact of the training ratios of the samples on the experimental results.

**Figure 14:**
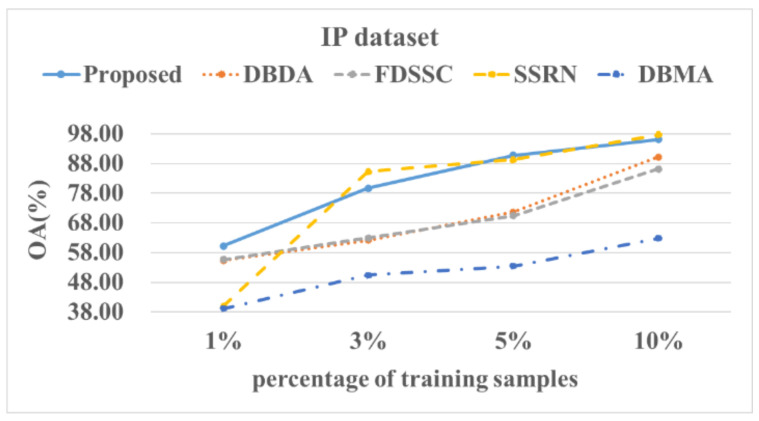
OA results of the different methods from various proportions of training samples using the IP dataset.

**Figure 15:**
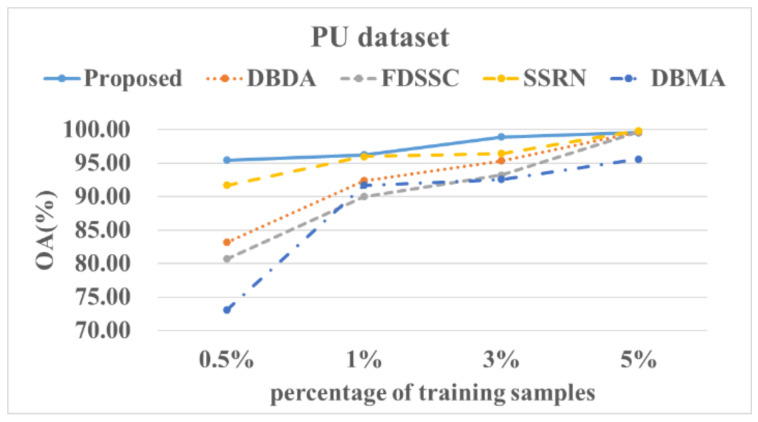
OA results of the different methods from various proportions of training samples using the PU dataset.

**Figure 16:**
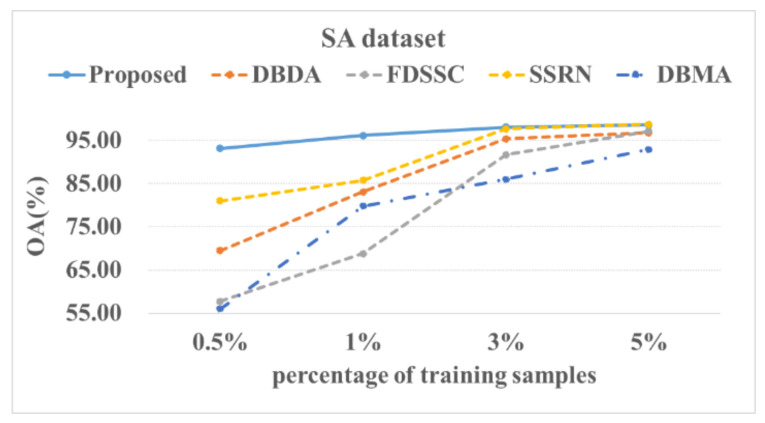
OA results of the different methods from various proportions of training samples using the SA dataset.

**Figure 17:**
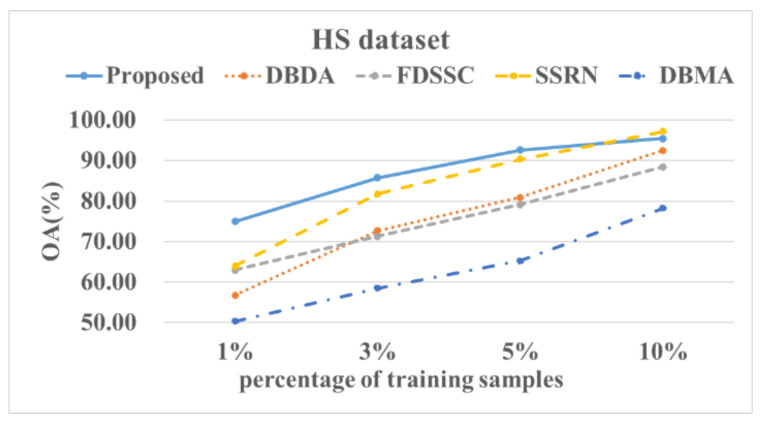
OA results of the different methods from various proportions of training samples using the HS dataset.

Figure 14, Figure 15, Figure 16 and Figure 17 show these experimental results. The accuracy increased with the number of samples. After the number of samples reached a certain point, the accuracy of several methods became similar. For the PU and SA datasets, we used 0.5%, 1%, 3%, and 5% of the data for the training samples. For the IP and HS datasets, we used 1%, 3%, 5%, and 10% of the data for the training samples. The DBMA method performed the worst. When the proportion of samples that was used for training was 1%, a 38% accuracy was achieved for the IP dataset. For the SA, PU, and HS datasets, the performances of the DBDA and FDSSC methods were almost equal. Overall, our proposed method demonstrated obvious advantages, especially when using a limited number of annotated samples.

## 5. Conclusions

Considering the limitations of the local characteristics of CNNs, we employed a transformer for HSI classification, which possessed the ability to perform long-range modeling. To overcome the time-consuming defects of the transformer, we committed to constructing a lightweight vision transformer, which was based on the MobileViT. Furthermore, we established a 3D-CAE model to reduce the data dimensionality and address the spectral redundancy of HSIs. Based on the above-mentioned structures, we proposed a lightweight HSI classification model named CAEVT. First, we carried out comparative experiments using four commonly used datasets and the proposed method achieved the best performance. Compared to the traditional PCA and LDA dimensionality reduction methods, the experimental results showed that 3D-CAE could extract features from the original HSIs more effectively by obtaining the nonlinear relationship between the bands. In addition, we conducted ablation studies and proved the effectiveness of the MobileViT structure in improving classification accuracy. Then, we compared and analyzed the number of parameters and the memory occupation of each method, which proved the lightweight nature and computational efficiency of the CAEVT network. Finally, we analyzed the impact of different proportions of training samples on the performance of the proposed method and the performance was better than that of the other methods for the different proportions of training samples, especially with a limited number of labeled training samples. Overall, the CAEVT network is effective and efficient. In the future, we plan to further explore the application of transformers in HSI classification.

## Figures and Tables

**Table 1:** Categories and sample numbers of the IP and SA datasets.

IP			SA		
	**Classes**	**Samples**		**Classes**	**Samples**
1	Alfalfa	46	1	Brocoli_green_weeds_1	2009
2	Corn-notill	1428	2	Brocoli_green_weeds_1	3726
3	Corn-mintill	830	3	Fallow	1976
4	Corn	237	4	Fallow_rough_plow	1394
5	Grass-pasture	483	5	Fallow_smooth	2678
6	Grass-trees	730	6	Stubble	3959
7	Grass-pasture-moved	28	7	Celery	3579
8	Hay-windrowed	478	8	Grapes_untrained	11,271
9	Oats	20	9	Soil_vinyard_develop	6203
10	Soybean-notill	972	10	Corn_senesced_green_weeds	3278
11	Soybean-mintill	2455	11	Lettuce_romaine_4wk	1068
12	Soybean-clean	593	12	Lettuce_romaine_5wk	1927
13	Wheat	205	13	Lettuce_romaine_6wk	916
14	Woods	1265	14	Lettuce_romaine_7wk	1070
15	Buildings-grass-trees-drives	386	15	Vinyard_untrained	7268
16	Stone-steel-towers	93	16	Vinyard_vertical_trellis	1807
	Total	10,249		Total	54,129

**Table 2:** Categories and sample numbers of the HS and PU datasets.

HS			PU		
	**Classes**	**Samples**		**Classes**	**Samples**
1	Healthy grass	1251	1	Asphalt	6631
2	Stressed grass	1254	2	Meadows	18,649
3	Synthetic grass	697	3	Gravel	2099
4	Tree	1244	4	Trees	3064
5	Soil	1242	5	Painted metal sheets	1345
6	Water	325	6	Bare soil	5029
7	Residential	1268	7	Bitumen	1330
8	Commercial	1244	8	Self-blocking bricks	3682
9	Road	1252	9	Shadows	947
10	Highway	1227			
11	Railway	1235			
12	Parking lot1	1233			
13	Parking lot2	469			
14	Tennis court	428			
15	Running track	660			
	Total	15,029		Total	42,776

**Table 3:** Parameter settings of the proposed 3D-CAE model when applied to the Pavia University dataset.

Layer (Type)	Input Size	Kernel	Stride	Output Size
Conv-3 × 3	(1, 103 × 9 × 9)	(24, 11 × 3 × 3)	(1 × 1 × 1)	(24, 93 × 7 × 7)
BN + PReLU	(24, 93 × 7 × 7)			(24, 93 × 7 × 7)
Conv-1 × 1	(24, 93 × 7 × 7)	(48, 11 × 1 × 1)	(1 × 1 × 1)	(48, 83 × 7 × 7)
BN + PReLU	(48, 83 × 7 × 7)			(48, 83 × 7 × 7)
Pooling	(48, 83 × 7 × 7)	(9 × 1 × 1)	(9 × 1 × 1)	(48, 9 × 7 × 7)
Deconv-1 × 1	(48, 9 × 7 × 7)	(24, 9 × 1 × 1)	(10 × 1 × 1)	(24, 89 × 7 × 7)
BN + PReLU	(24, 89 × 7 × 7)			(24, 89 × 7 × 7)
Deconv-3 × 3	(24, 89 × 7 × 7)	(1, 15 × 3 × 3)	(1 × 1 × 1)	(1, 103 × 9 × 9)
BN	(1, 103 × 9 × 9)			(1, 103 × 9 × 9)

**Table 4:** Parameter settings of the MobileViT block when applied to the Pavia University dataset.

Layer (Type)	Input Size	Output Size
Conv-3 × 3	(32 × 4 × 4)	(32 × 4 × 4)
BN + SiLU	(32 × 4 × 4)	(32 × 4 × 4)
Conv-1 × 1	(32 × 4 × 4)	(8 × 4 × 4)
BN + SiLU	(8 × 4 × 4)	(8 × 4 × 4)
Rearrange	(8 × 4 × 4)	(4 × 4 × 8)
Transformer	(4 × 4 × 8)	(4 × 4 × 8)
Rearrange	(4 × 4 × 8)	(8 × 4 × 4)
Conv-1 × 1	(8 × 4 × 4)	(32 × 4 × 4)
BN + SiLU	(32 × 4 × 4)	(32 × 4 × 4)
Fusion	(2, 32 × 4 × 4)	(64 × 4 × 4)
Conv-3 × 3	(64 × 4 × 4)	(32 × 4 × 4)
BN + SiLU	(32 × 4 × 4)	(32 × 4 × 4)

**Table 5:** Parameter settings of the CAEVT network when applied to the Pavia University dataset.

Layer (Type)	Input Size	Output Size
3D-CAE	(1, 103 × 9 × 9)	(48, 9 × 7 × 7)
Reshape	(48, 9 × 7 × 7)	(432 × 7 × 7)
Conv-3 × 3	(432 × 7 × 7)	(32 × 4 × 4)
BN + SiLU	(32 × 4 × 4)	(32 × 4 × 4)
MobileViT	(32 × 4 × 4)	(32 × 4 × 4)
Conv-1 × 1	(32 × 4 × 4)	(16 × 4 × 4)
BN + SiLU	(16 × 4 × 4)	(16 × 4 × 4)
Reshape	(16 × 4 × 4)	(1 × 256)
Linear	(1 × 256)	(1 × 9)

**Table 6:** Categorized results for the IP dataset.

Class	SSRN	FDSSC	DBMA	DBDA	Proposed
1	0.00	0.00	0.00	0.00	**100.00**
2	**96.02**	44.92	68.70	47.98	89.86
3	77.44	61.43	23.22	86.89	**87.82**
4	**100.00**	0.00	0.00	**100.00**	77.90
5	95.67	87.05	62.93	92.04	**99.29**
6	**99.85**	77.45	62.96	86.15	94.97
7	0.00	0.00	0.00	0.00	**100.00**
8	**91.88**	90.34	93.38	89.40	90.66
9	0.00	0.00	0.00	0.00	**87.50**
10	82.47	79.55	**91.57**	82.56	86.98
11	89.89	91.54	43.81	60.94	**90.02**
12	72.54	74.55	75.00	**85.86**	84.65
13	**92.46**	0.00	63.83	96.81	88.29
14	**95.52**	88.04	80.29	89.35	95.27
15	90.45	48.28	62.42	93.38	**95.22**
16	**100.00**	**100.00**	98.73	93.90	**100.00**
OA (%)	89.27	70.43	53.49	71.66	**90.71**
AA (%)	68.56	50.65	40.92	57.14	**78.61**
Kappa × 100	87.73	66.29	44.91	66.84	**89.37**

**Table 7:** Categorized results for the SA dataset.

Class	SSRN	FDSSC	DBMA	DBDA	Proposed
1	98.85	**100.00**	**100.00**	98.75	97.50
2	92.65	58.25	91.03	**100.00**	99.65
3	91.90	44.86	85.79	88.86	**96.37**
4	93.94	93.67	96.93	97.58	**98.77**
5	91.56	68.54	88.42	68.59	**100.00**
6	**100.00**	99.95	99.92	**100.00**	99.87
7	96.43	98.73	95.61	98.59	**99.94**
8	**93.75**	61.22	60.16	73.16	91.83
9	78.42	63.30	93.62	83.51	**99.22**
10	53.75	60.52	87.43	93.10	**99.06**
11	0.00	0.00	54.99	0.00	**99.51**
12	0.00	92.75	87.62	65.96	**96.47**
13	**100.00**	**100.00**	95.33	99.49	99.78
14	84.90	61.46	85.69	**100.00**	99.23
15	80.54	31.70	52.62	67.36	**89.14**
16	**100.00**	**100.00**	94.87	**100.00**	99.61
OA (%)	85.77	68.77	79.84	83.11	**96.22**
AA (%)	75.31	58.31	83.98	80.05	**97.77**
Kappa × 100	84.10	64.61	77.37	81.06	**95.79**

**Table 8:** Categorized results for the PU dataset.

Class	SSRN	FDSSC	DBMA	DBDA	Proposed
1	**97.38**	77.93	89.85	82.00	97.02
2	**97.22**	97.84	92.99	96.26	95.22
3	79.69	**100.00**	80.56	**100.00**	87.03
4	99.83	98.07	97.52	98.54	**99.92**
5	**100.00**	99.62	99.84	**100.00**	**100.00**
6	98.46	96.47	93.79	96.70	**99.79**
7	87.44	**100.00**	93.37	99.69	99.52
8	93.19	67.20	87.49	80.04	**95.93**
9	95.96	**99.32**	79.04	99.02	97.89
OA (%)	96.01	90.00	91.68	92.41	**96.25**
AA (%)	94.47	75.61	87.32	82.95	**94.60**
Kappa × 100	94.69	86.61	88.83	89.83	**94.98**

**Table 9:** Categorized results for the HS dataset.

Class	SSRN	FDSSC	DBMA	DBDA	Proposed
1	87.89	83.23	84.86	81.74	**96.42**
2	86.05	69.23	59.81	70.95	**91.61**
3	98.89	81.24	68.20	80.48	**99.36**
4	**95.82**	86.18	68.89	76.68	94.01
5	97.69	96.31	95.81	97.23	**98.48**
6	92.99	92.49	60.47	**100.00**	96.76
7	83.05	69.64	64.82	70.36	**86.76**
8	89.69	**94.14**	73.59	91.51	88.67
9	76.62	67.84	54.73	68.51	**84.04**
10	95.67	74.01	67.21	84.31	**95.26**
11	**95.02**	66.52	42.28	86.62	92.92
12	88.60	88.29	64.00	82.27	**91.83**
13	**92.90**	77.69	80.00	89.53	85.81
14	99.47	95.01	68.31	88.57	**99.73**
15	**98.40**	94.39	71.43	81.42	97.97
OA (%)	90.33	79.17	65.33	80.99	**92.67**
AA (%)	88.92	78.47	63.65	80.62	**90.78**
Kappa × 100	89.54	77.46	62.48	79.43	**92.06**

**Table 10:** Influence of different activation functions.

	PReLU	Tanh	Sigmoid	Proposed
OA (%)	94.40	94.85	95.53	96.25
AA (%)	90.61	92.06	92.98	94.60
Kappa × 100	92.54	93.15	94.04	94.98

**Table 11:** Training and testing time consumption for the IP dataset.

Dataset	Algorithm	Training Time(s)	Testing Time(s)
IP	SSRN	219.51	1.63
	FDSSC	526.31	1.89
	DBMA	67.89	1.42
	DBDA	63.36	2.00
	Proposed	28.43	2.84

**Table 12:** Training and testing time consumption for the PU dataset.

Dataset	Algorithm	Training Time(s)	Testing Time(s)
PU	SSRN	181.45	4.00
	FDSSC	386.93	4.75
	DBMA	51.24	5.11
	DBDA	43.34	4.74
	Proposed	24.70	9.35

**Table 13:** Training and testing time consumption for the SA dataset.

Dataset	Algorithm	Training Time(s)	Testing Time(s)
SA	SSRN	270.78	9.25
	FDSSC	540.10	9.82
	DBMA	95.87	7.77
	DBDA	82.24	10.56
	Proposed	30.53	13.19

**Table 14:** Training and testing time consumption for the HS dataset.

Dataset	Algorithm	Training Time(s)	Testing Time(s)
HS	SSRN	314.31	1.87
	FDSSC	61.63	2.16
	DBMA	64.12	1.39
	DBDA	59.86	2.06
	Proposed	40.53	3.61

## Data Availability

The datasets that are involved in this paper are all public datasets.

## Abbreviations

The following abbreviations are used in this manuscript:

SSRN	Spectral–spatial residual network
FDSSC	Fast dense spectral–spatial convolution
DBMA	Double-branch multi-attention mechanism network
DBDA	Double-branch dual-attention mechanism network
